# Brain signatures of nociplastic pain: Fibromyalgia Index and descending modulation at population level

**DOI:** 10.1093/brain/awaf307

**Published:** 2025-08-17

**Authors:** Eoin Maurice Kelleher, Frederik Lange, Vishvarani Wanigasekera, Trishna Rathod-Mistry, Thomas Nichols, Ben Seymour, Irene Tracey, Andrew Reilly Segerdahl, Anushka Irani

**Affiliations:** Oxford University Centre for Integrative Neuroimaging, FMRIB, Nuffield Department of Clinical Neurosciences, University of Oxford, Oxford OX3 9DU, UK; Department of Anesthesia, Critical Care and Pain Medicine, Massachusetts General Hospital, Boston, MA 02114, USA; Oxford University Centre for Integrative Neuroimaging, FMRIB, Nuffield Department of Clinical Neurosciences, University of Oxford, Oxford OX3 9DU, UK; Oxford University Centre for Integrative Neuroimaging, FMRIB, Nuffield Department of Clinical Neurosciences, University of Oxford, Oxford OX3 9DU, UK; Nuffield Department of Orthopaedics, Rheumatology and Musculoskeletal Sciences, University of Oxford, Oxford OX3 7LD, UK; Oxford University Centre for Integrative Neuroimaging, FMRIB, Nuffield Department of Clinical Neurosciences, University of Oxford, Oxford OX3 9DU, UK; Big Data Institute, Nuffield Department of Population Health, University of Oxford, Oxford OX3 7LF, UK; Oxford University Centre for Integrative Neuroimaging, FMRIB, Nuffield Department of Clinical Neurosciences, University of Oxford, Oxford OX3 9DU, UK; Oxford University Centre for Integrative Neuroimaging, FMRIB, Nuffield Department of Clinical Neurosciences, University of Oxford, Oxford OX3 9DU, UK; Oxford University Centre for Integrative Neuroimaging, FMRIB, Nuffield Department of Clinical Neurosciences, University of Oxford, Oxford OX3 9DU, UK; Oxford University Centre for Integrative Neuroimaging, FMRIB, Nuffield Department of Clinical Neurosciences, University of Oxford, Oxford OX3 9DU, UK; Nuffield Department of Orthopaedics, Rheumatology and Musculoskeletal Sciences, University of Oxford, Oxford OX3 7LD, UK; Division of Rheumatology, Mayo Clinic Florida, Jacksonville, FL 32224, USA

**Keywords:** chronic pain, nociplastic pain, fibromyalgia, descending pain modulation system, functional MRI, population neuroimaging

## Abstract

Nociplastic pain is defined by altered nociceptive processing in the absence of clear peripheral damage or somatosensory lesions. The Fibromyalgia Index (FMI), derived from the 2016 diagnostic criteria, is increasingly used as a marker of nociplastic pain severity in clinical studies, yet its neurobiological validity remains untested at scale.

Using multimodal neuroimaging data from over 40 000 participants in UK Biobank, we examined whether FMI scores were associated with altered functional and structural connectivity within the descending pain modulatory system (DPMS), a brain network involved in endogenous pain control and implicated in nociplastic pain conditions. Functional connectivity was assessed using resting-state functional MRI (rfMRI), and structural connectivity using diffusion-weighted MRI (dMRI) tractography. Connectivity was quantified between seven DPMS regions: periaqueductal grey (PAG), rostral ventromedial medulla (RVM), hypothalamus, amygdala, rostral and subgenual anterior cingulate cortex (rACC, sgACC), and dorsolateral prefrontal cortex (dlPFC). Multi-group structural equation models tested associations between FMI scores and connectivity, stratified by chronic pain status. Mediation models evaluated which aspects of nociplastic pain accounted for the observed associations: widespread pain and SPACE symptoms (sleep disturbance, pain, affect, cognitive problems, and low energy). To assess specificity, we repeated analyses using the Douleur Neuropathique 4 (DN4), a measure of neuropathic pain, and average pain intensity as comparison outcomes.

In 22 139 individuals with chronic pain (58% female; mean age 64.8, standard deviation 7.59), FMI scores were associated with altered structural connectivity between the PAG and amygdala [β = 0.023, 95% confidence interval (CI): 0.0087 to 0.039; *P*_corr_ = 0.0125] and between the PAG and hypothalamus (β = −0.029, 95% CI: −0.043 to −0.015; *P*_corr_ = 0.0013). Functional connectivity in the same circuits showed smaller effects. These associations were not observed in individuals without chronic pain. Mediation analyses revealed that PAG-amygdala and PAG-hypothalamus connectivity were partially explained by fatigue, sleep duration, and widespread pain. DPMS connectivity was not significantly associated with neuropathic pain or average pain intensity.

These findings suggest that FMI scores reflect biologically meaningful changes in brain connectivity, particularly in subcortical DPMS circuits implicated in affective and homeostatic dimensions of pain. Structural connectivity was more strongly associated with FMI than functional measures, possibly reflecting cumulative effects of chronic pain on white matter architecture. The absence of similar associations for other pain outcomes supports the specificity of FMI as a marker of nociplastic pain severity. These results provide a neurobiological basis for the FMI and support its use in population research and biomarker development for nociplastic pain.

## Teaser

Kelleher *et al*. analysed brain scans from over 40,000 people and found that higher fibromyalgia scores—used to measure nociplastic pain—were associated with changes in brain circuits involved in pain control, supporting their use as a marker of this type of pain in the general population.

## Introduction

Nociplastic pain, a relatively recent addition to the pain taxonomy, is defined by altered nociceptive processing in the absence of clear peripheral damage or somatosensory lesions.^1^ Despite increasing recognition, there remains uncertainty surrounding its clinical boundaries, underlying mechanisms, and how best to identify individuals with this pain phenotype, particularly at a population level.

Conditions such as irritable bowel syndrome, endometriosis and tension-type headache often exhibit nociplastic pain features, although fibromyalgia is often regarded as the archetypal example.^2^ These conditions are not defined by a single pain mechanism; rather, nociplastic pain may coexist with nociceptive and/or neuropathic processes, contributing to overall symptom burden. Increasingly, these disorders are understood to lie along a continuum of shared symptomatology and shared central mechanisms, and are collectively referred to as chronic overlapping pain conditions.^3^

Nociplastic pain is characterized by widespread pain and a constellation of somatic symptoms that extend beyond pain alone. These include fatigue, poor sleep, cognitive dysfunction, mood disturbances and sensory sensitivity—a symptom constellation captured by the SPACE cluster (sleep disturbance, pain, affect, cognitive dysfunction, and low energy).^4^

Dysfunctional pain modulation, in particular through impaired descending inhibition and/or enhanced facilitation of nociceptive signals, is thought to underlie these clinical manifestations.^2^ The descending pain modulatory system (DPMS) is central to this process, comprising a distributed network of brain regions including the periaqueductal grey (PAG), rostral ventromedial medulla (RVM), hypothalamus, amygdala, anterior cingulate cortex (ACC) and dorsolateral prefrontal cortex (dlPFC).^5,6^ The DPMS receives inputs from higher-order cortical regions and modulates spinal nociceptive transmission via descending projections.^5,7^ Structural and functional abnormalities within the DPMS have been reported across several chronic pain conditions, including fibromyalgia, yet few studies have evaluated these associations at the population level.^8-10^

Recent theoretical models have conceptualized nociplastic pain as an aberrant homeostatic state, wherein the brain overestimates peripheral injury and generates maladaptive behavioural and physiological responses.^11^ This state is thought to involve excessive descending sensitization, as well as exaggerated defensive (e.g. anxiety, sleep disruption) and recuperative behaviours (e.g. fatigue, low mood). While such responses are adaptive in the context of actual tissue damage, they become maladaptive when injury is inferred inaccurately. This framework offers a mechanistic link between generalized pain and broader symptoms affecting motivation, emotion and sleep. It also predicts altered functional and structural connectivity in brainstem-limbic-prefrontal circuits, particularly involving the PAG, hypothalamus, amygdala and ventromedial prefrontal cortex.^12^ Understanding how these circuits are disrupted in individuals with nociplastic pain could offer insight into both the pain experience and its broader psychosomatic impact.

The Fibromyalgia Index (FMI), derived from the 2016 American College of Rheumatology diagnostic criteria,^13^ may provide a continuous measure of nociplastic pain severity by combining the Widespread Pain Index (WPI) and Symptom Severity Scale (SSS). It is widely used in clinical and research settings, showing predictive value for pain outcomes in the general population, such as following surgery.^14^ Although originally designed to support fibromyalgia diagnosis, the FMI can also be used as a dimensional tool to capture nociplastic features across other pain conditions, regardless of formal diagnostic classification. However, the neurobiological validity of the FMI remains uncertain, and whether it corresponds to objective changes in pain-modulatory brain networks has not been tested in large-scale population-based studies. Furthermore, it is unclear which specific features of nociplastic pain, such as those captured by the SPACE symptom cluster or widespread pain, account for this relationship.

In this study, we aimed to evaluate the neurobiological relevance of the FMI using multimodal neuroimaging data from UK Biobank, a large population cohort. Specifically:

- Objective 1: To investigate the association between functional (a) and structural (b) connectivity in the DPMS with FMI scores in adults with and without chronic pain.
- Objective 2: To evaluate which characteristics of nociplastic pain, including SPACE symptoms and widespread pain, account for the relationship between key connectivity pairs in the DPMS and FMI score.
- Objective 3: To evaluate whether observed connectivity-FMI associations are specific to nociplastic pain, or instead reflect general pain severity or neuropathic pain, using the Douleur Neuropathique 4 (DN4) and average pain intensity as comparison outcomes.

Finally, as an exploratory analysis, we examined whether associations between DPMS connectivity and FMI varied across age groups.

Understanding the neural correlates of the FMI may clarify its role as a clinically meaningful measure of nociplastic pain and support its application in both research and practice.

## Materials and methods

### Study population

This cross-sectional study is nested within UK Biobank, a population-based cohort of ∼500 000 adults aged 40–69, recruited between 2006 and 2010.^15^ Participants provided sociodemographic, lifestyle and health data at baseline, and were later invited to follow-up assessments including neuroimaging and online questionnaires. UK Biobank aims to image 100 000 participants across four centres (Stockport, Newcastle, Reading and Bristol). Invitations were initially emailed to ∼330 000 participants, with postal invitations sent in 2020; fewer than 0.5% were ineligible or uncontactable.^16^

**Figure 1:**
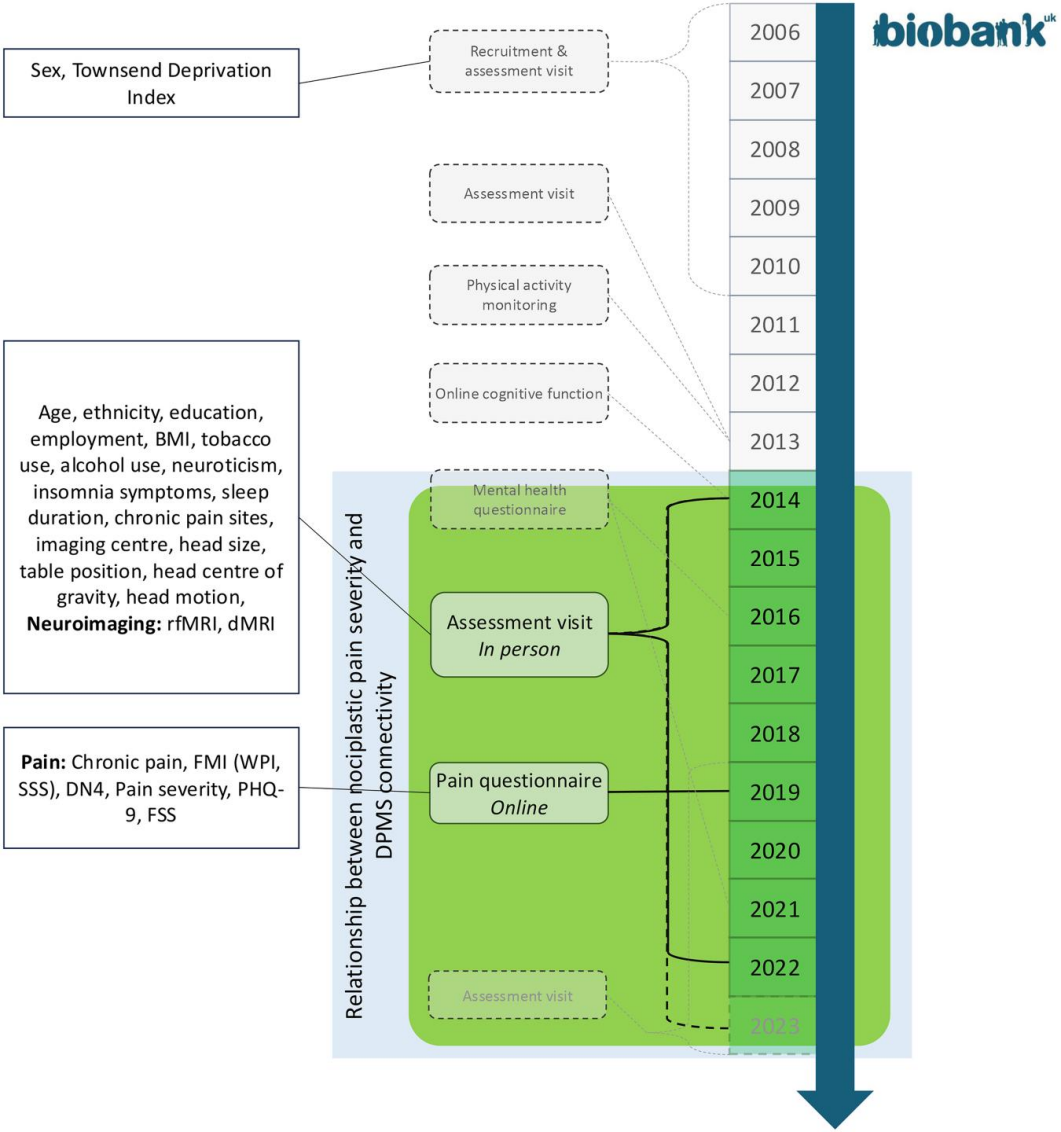
**Flow diagram of the timeline for the main assessments in UK Biobank**. The assessments used in the current study are highlighted in the shaded box. All participants for whom UK Biobank have a current email address (*N* = ∼333 000) were invited to attend follow-up visits and complete online questionnaires. Commencing 2020, UK Biobank also began sending postal invitations for imaging, in addition to email invitations. A small number (<0.5%) of participants have withdrawn or moved outside the UK. Functional connectivity (rfMRI) and structural connectivity (dMRI) data were extracted for participants scanned between May 2014 and June 2023. Sex and Townsend Deprivation Index were only assessed at recruitment. BMI = body mass index; rfMRI = resting-state functional MRI; dMRI = diffusion-weighted MRI; NRS = numeric rating scale; FMI = Fibromyalgia Index; DN4 = Douleur Neuropathique 4; PHQ-9 = Patient Health Questionnaire 9-item; FSS = Fatigue Severity Scale.

For this study, we included participants who attended neuroimaging between 2014 and 2023 and completed the 2019 Pain Questionnaire (Fig. 1). Imaging was paused from March 2020 to February 2021 due to coronavirus disease 2019. Participants with dementia or serious neurological conditions affecting pain reporting were excluded. The UK Biobank received ethical approval from the NHS National Research Ethics Service (Ref. 11/NW/0382); this study was approved under application 45 465.

### Behavioural data

In this study, we used the FMI score as a measure of nociplastic severity in Objectives 1 and 2 to find any associations between functional (using resting-state imaging) and structural (using diffusion-weighted imaging) connectivity patterns between key nodes in the DPMS. Chronic pain status was determined by self-report, defined as pain lasting more than 3 months, in line with International Association for the Study of Pain (IASP) criteria^1^ and based on participants’ responses to the 2019 UK Biobank pain questionnaire. The FMI, derived from the 2016 Fibromyalgia Survey Criteria, which combines the WPI (0–19) and SSS (0–12), creating a continuous measure (0–31) where higher scores indicate more severe nociplastic pain.^13^

In Objective 3, we investigated whether the associations identified between DPMS connectivity and nociplastic pain severity were specific to nociplastic pain, or instead reflected broader dimensions of pain, such as pain intensity or neuropathic pain features. To do this, we repeated the analyses using two alternative outcome measures. First, we used the DN4 questionnaire, a validated measure of neuropathic pain symptoms.^17^ Second, we used average pain intensity, assessed on a 0–10 numeric rating scale (NRS), as a general measure of pain severity. These replication analyses employed the same approach as for the primary FMI outcome, allowing us to determine whether the observed connectivity patterns were specific to nociplastic pain or more generally related to other pain phenotypes.

Socio-demographic and lifestyle confounds were selected *a priori*,^2^ and consisted of age and sex (male, female), ethnicity (white, non-white), Townsend index of material deprivation,^18^ and education (university degree, no degree), tobacco use (current or never/former), and body mass index (BMI, kg/m^2^). Continuous variables were centred to a mean of zero.

### Neuroimaging data

From the UK Biobank imaging visit, functional and structural connectivity in the DPMS was measured using multimodal neuroimaging.^19^ Imaging was performed on Siemens Skyra 3T scanners across four dedicated centres. This study used preprocessed rfMRI and diffusion weighted MRI (dMRI) data generated by the UK Biobank image-processing pipeline.^19-21^

#### Resting-state functional MRI

We analysed rfMRI data acquired during participants’ first imaging visit, restricting analyses to phase 3 onwards to ensure protocol consistency.^20^ Earlier phases, comprising approximately 500 participants, were excluded due to differing scan protocols. Each rfMRI scan lasted 6 min and yielded 490 time points, with a spatial resolution of 2.4 × 2.4 × 2.4 mm, a repetition time (TR) of 0.735 s and an echo time (TE) of 39 ms.

#### Data preprocessing

Preprocessing was previously performed by UK Biobank and followed their standard pipeline.^20^ Motion correction was performed using MCFLIRT, followed by grand-mean intensity normalization across the 4D dataset. A high-pass temporal filter (σ = 50 s) was applied to remove low-frequency signal drift. Spatial distortions were corrected using both EPI unwarping and gradient distortion correction. Denoising was carried out using independent components analysis (ICA) via FMRIB's ICA-based X-noiseifier (FIX),^22,23^ which had been trained on 40 hand-labelled UK Biobank datasets. Functional images were registered to the corresponding T1-weighted structural scans using boundary-based registration (BBR), with subsequent transformation to Montreal Neurological Institute 152 (MNI152) standard space. No low-pass filtering or spatial smoothing was applied. Quality control included automated checks (e.g. framewise displacement thresholds) and visual inspection of a subset of images to confirm the integrity of preprocessing and alignment.

### Definition of region of interest masks

Binary masks were created for seven regions of interest (ROIs) central to the DPMS: the rostral ventromedial medulla (RVM), periaqueductal grey (PAG), hypothalamus, amygdala, rostral anterior cingulate cortex (rACC), subgenual ACC (sgACC) and dorsolateral prefrontal cortex (dlPFC).

The RVM mask was manually drawn in FSLeyes using anatomical landmarks from Duvernoy's Atlas of the Human Brainstem and Cerebellum, guided by the location of the nucleus raphe magnus and adjacent nucleus reticularis gigantocellularis.^24-26^ The PAG mask was similarly hand-drawn based on the approach of Ezra *et al*.,^27^ cross-referenced with Duvernoy's Atlas for anatomical accuracy.

The hypothalamus mask was created with reference to the MRI-based atlas by Baroncini *et al*.,^28^ using landmarks such as the optic chiasm, mammillary bodies and third ventricle. Amygdala masks were derived from the Harvard-Oxford Atlas, thresholded at 50% to include voxels with high probability of belonging to the structure.^29-32^

For rACC and sgACC, voxels from the anterior cingulate cortex in the Harvard-Oxford Atlas (thresholded at 50%) were subdivided using histological boundaries defined by Vogt *et al*.^33^ Grey matter-only masks were applied using FSL's FAST to exclude non-neural tissue.^34^

The dlPFC mask was constructed by combining atlas-defined Brodmann areas 8, 9, 46 and 9/46, also thresholded at 50% and restricted to grey matter.^35^

All masks were generated in MNI152 1 mm standard space for accuracy and transformed to MNI152 2 mm (accounting for partial volume effects) using FLIRT,^36^ ensuring compatibility with downstream analyses (Table 1 and Supplementary Fig. 1).

**Table 1:** Summary of how binary ROI masks in the DPMS were derived

ROI mask	Key sources	Description
RVM	Duvernoy's Atlas of the Human Brainstem and Cerebellum (Naidich *et al*.^26^)	Hand drawn in FSLeyes using Duvernoy's Atlas in reference to nucleus raphe magnus, nucleus gigantocellularis and facial nucleus.
PAG	Ezra *et al*.,^27^ Duvernoy's Atlas of the Human Brainstem and Cerebellum (Naidich *et al*.^26^)	Derived from work by Ezra *et al*.,^27^ who delineated the PAG using diffusion MRI and the B0 image, cross-referenced with Duvernoy's Atlas for accurate boundary definition.
Hypo	Baroncini *et al*.,^28^ Duvernoy's Atlas of the Human Brainstem and Cerebellum (Naidich *et al*.^26^)	Created using Baroncini *et al*.'s histological and MRI-based approach^28^ with landmarks like the optic chiasm and mammillary bodies; boundaries refined using Duvernoy's Atlas.
Amyg	Harvard-Oxford Atlas, Frazier *et al*.,^29^ Desikan *et al*.,^30^ Makris *et al*.,^31^ Goldstein *et al.*^32^	Derived from the Harvard-Oxford Atlas with a 50% probability threshold to include voxels highly likely to belong to the amygdala.
rACC	Vogt *et al*.,^33^ Harvard-Oxford Atlas	Based on Vogt *et al*.^33^ histological delineation of the ACC and the Harvard-Oxford Atlas, thresholded at 50% probability and refined with grey matter mask.
sgACC	Vogt *et al*.,^33^ Harvard-Oxford Atlas	Developed using Vogt *et al*.^33^ ACC delineation combined with the Harvard-Oxford Atlas, with a 50% probability threshold applied and refined with grey matter mask.
dlPFC	Harvard-Oxford Atlas; Brodmann Areas (8, 9, 46, 9/46); Cieslik *et al*.^35^	Constructed by combining Harvard-Oxford regions corresponding to Brodmann Areas 8, 9, 46 and 9/46, thresholded at 50% probability and refined with a grey matter mask.

All regions of interest (ROIs) are bilateral and in 2 mm MNI152 space. Amyg = amygdala; dlPFC = dorsolateral prefrontal cortex; DPMS = descending pain modulatory system; Hypo = hypothalamus; PAG = periaqueductal grey; rACC = rostral anterior cingulate cortex; RVM = rostral ventromedial medulla; sgACC = subgenual anterior cingulate cortex.

#### Estimation of functional connectivity matrices

For each participant, the mean preprocessed 4D time series within each ROI was extracted using FSL's *fslmeants*. Participants with incomplete scans (less than or more then 490 time points) or missing ROI data were excluded. Time series were preprocessed in R using a custom function based on *nets_load* from FSLnets, including demeaning, variance-normalization and concatenation for group-level analyses. Spectral analysis (adapted from *nets_spectra*) was performed to assess time-series quality; mean power spectra were averaged and visually inspected.

Functional connectivity matrices were computed using partial correlations between the seven ROIs (21 unique pairs), following the *netmats* approach. The inverse covariance matrix was computed, normalized by its diagonal, and the diagonal elements were zeroed. Fisher's *R*-to-*z* transformation was applied. Quality checks included matrix inversion diagnostics and filtering of invalid values. Connectivity values were standardized (mean = 0, SD = 1) and winsorized at ±3 SD to limit outlier influence without removal.^37^

For the analysis, six PAG connectivity edges were examined: RVM-PAG, PAG-hypothalamus, PAG-amygdala, PAG-rACC, PAG-sgACC and PAG-dlPFC (Fig. 1).

### Diffusion-weighted MRI

Structural connectivity between key DPMS nodes—RVM, PAG, amygdala and hypothalamus—was estimated using probabilistic tractography on dMRI data. This method models uncertainty in fibre orientation to map likely white matter pathways, allowing for complex tract reconstruction.^38^

#### Preprocessing

Preprocessing was conducted by UK Biobank, and is described elsewhere.^39^ In brief, diffusion MRI data underwent eddy current and motion correction using FSL's *eddy*,^40^ which also included outlier slice replacement.^41^ Gradient distortion correction was subsequently applied. Multiple b = 0 images were acquired throughout the diffusion sequence and were aligned and averaged to produce a single mean b = 0 image. This averaged image was used to generate a brain mask using BET, delineating brain tissue for tractography, and served as the reference for subsequent alignment and distortion correction. Pre-processed diffusion volumes, including the corrected b = 0 images, were merged into a single 4D file for downstream use. To enable spatial alignment, transformation matrices between dMRI and MNI152 space were computed using FSL's *convertwarp* and *invwarp*, allowing mapping of tractography results across participants.

#### Tractography

Probabilistic tractography was run with FSL's *probtrackx2*, estimating structural connections between seven ROIs from four nodes in the lower DPMS: RVM, PAG, left/right amygdala and left/right hypothalamus.^38^ Cortical regions (ACC, dlPFC) were excluded due to computational constraints. ROI seed masks were defined in MNI standard space and were transformed into each participant's diffusion space during tractography using subject-specific warp fields. A midline exclusion mask prevented spurious interhemispheric tracts beyond the corpus callosum. Parameters included 5000 samples per voxel, 0.5 mm step length and a 0.2 radian curvature threshold.

For each participant, region-pair connectivity values were extracted, symmetric edges (e.g. PAG-RVM and RVM-PAG) were averaged, and self-connections were excluded. Left/right connectivity pairs were averaged due to similar associations across hemispheres. Final analyses focused on six structural connections: RVM-PAG, RVM-hypothalamus, RVM-amygdala, PAG-hypothalamus, PAG-amygdala and hypothalamus-amygdala. All values were mean-centred, standardized and winsorized (±3 SD), as described for functional data.

#### Mediators

Nociplastic pain disorders are characterized by a symptom cluster consisting of sleep difficulties, pain, affect (depression/anxiety), cognitive problems (brain-fog), and low energy (fatigue). These have been termed ‘SPACE’ symptoms.^4^

The SPACE Cluster is defined as^4^: sleep duration (<7, 7–9, >9 h^42^), pain intensity (0–10 NRS), depressive symptoms (Patient Health Questionnaire 9-item, PHQ-9), anxiety symptoms (Generalized Anxiety Disorder 7-item, GAD-7), cognitive symptoms (subjective cognitive difficulties from SSS), and fatigue (Fatigue Severity Scale, FSS). Our rationale was to dissect nociplastic pain, as reflected by the FMI, into its underlying symptom domains and to test whether associations with DPMS connectivity could be explained by specific facets of the nociplastic phenotype. To reduce measurement overlap with the FMI, we selected alternative instruments for overlapping domains where possible (e.g. FSS instead of the SSS fatigue item).

WPI was separately considered as a mediator.

### Statistical analysis

Baseline characteristics were summarized using means and standard deviation (SD) for continuous variables, and frequencies with percentages for categorical variables for all participants and stratified by chronic pain status. Structural equation modelling (SEM) was used for all study objectives.

#### Objective 1: relationship between DPMS connectivity and nociplastic pain severity

Multi-group SEM was used to examine the association between functional (using rfMRI) and structural (using dMRI) DPMS connectivity edges with FMI, stratified by chronic pain status.

To assess whether DPMS connectivity collectively explained significant variance in FMI, we conducted an omnibus F-test comparing full and reduced linear models that included or excluded the six predefined PAG-related connectivity edges. To assess whether the overall pattern of associations between DPMS connectivity and FMI differed between participants with and without chronic pain, we compared constrained and unconstrained multi-group SEMs using a likelihood ratio test (LRT). Finally, to identify specific connectivity edges whose associations with FMI differed significantly between groups, Wald tests were conducted on each individual path coefficient.

#### Objective 2: mediation with SPACE symptoms and widespread pain

To examine whether SPACE symptoms and widespread pain mediated the relationship between DPMS connectivity and FMI scores, we fitted SEM models with SPACE and WPI scores as mediators, and FMI as the outcome. Predictors included DPMS edges identified as statistically significant in Objective 1. The mediators were the WPI and six SPACE symptom measures: sleep duration, pain intensity, fatigue, depression, anxiety, and cognitive difficulties. Separate mediation models were carried out for functional and structural connectivity, limited to individuals with chronic pain.

Our primary analysis modelled the six SPACE symptoms as parallel mediators to identify which symptom domains contributed most strongly to indirect effects. This approach allowed us to examine the multidimensional nature of nociplastic pain and clarify symptom-specific pathways linking brain connectivity to overall FMI scores. To account for intercorrelation between symptoms, residual covariances among mediators were freely estimated. WPI was included as a separate mediator to assess effects of widespread pain independently of SPACE symptoms.

As a sensitivity analysis, we also tested an alternative SEM in which SPACE symptoms loaded onto a single latent factor, capturing their shared variance. This complementary approach tested whether a general symptom burden dimension accounted for brain-FMI associations. Multicollinearity between mediators was assessed using the variance inflation factor (VIF).

Indirect effects were computed as the product of path coefficients from DPMS connectivity to mediators and from mediators to FMI.^43^ Total effects were the sum of direct and indirect paths. Where mediation was of interest, bias-corrected and accelerated bootstrapping with 1000 resamples was used to estimate confidence intervals and *P*-values.^44^

#### Objective 3: replication in other pain phenotypes

To determine whether findings from Objective 1 generalized to other pain types, the same analyses from Objective 1 were repeated for DN4,^17^ a measure of neuropathic pain symptoms, and average pain intensity on a numeric rating scale.

#### Exploratory analysis: age-related variation in connectivity effects

To examine whether associations between DPMS connectivity and FMI varied by age, we conducted exploratory multi-group SEMs stratified into 5-year age bands (from 40 to 70 years) in participants with chronic pain. Functional and structural connectivity models were fitted separately, and unconstrained group-specific path estimates were compared using LRTs. Wald tests were applied to evaluate group differences in each connectivity path.

#### Model estimation and confounder adjustment

All models were adjusted for imaging and lifestyle and socio-demographic confounders. Imaging confounders were based on the UK Biobank imaging confound guidelines,^45^ and included: scan date, head size, table position (head centre of gravity on the *x*, *y*, *z* axes) and mean head motion during rfMRI (motion estimates for dMRI were not available). Lifestyle and socio-demographic confounding variables were selected based on their *a priori* hypothesized associations with nociplastic pain^2^ and included: age, sex, education level (degree versus no degree), Townsend Deprivation Index, self-reported ethnicity (white versus non-white), smoking status (current versus non-smoker) and BMI.

Models were estimated using maximum likelihood (ML) in R (v4.4.1) with the *lavaan* package. Analyses were restricted to complete cases. Parameter estimates are reported as standardized coefficients. Model assumptions (linearity, multivariate normality, identification) were checked and found acceptable. Model fit was assessed using the Comparative Fit Index (CFI), Tucker-Lewis Index (TLI) and root mean square error of approximation (RMSEA), with conventional cut-offs used to determine adequate fit. Two-sided *P* values <0.05 were considered statistically significant. *P*-values were adjusted for multiple comparisons using the false discovery rate (FDR) correction (Benjamini–Hochberg procedure).^46^ Field ID codes for UK Biobank variables are provided in Supplementary Table 1. The study follows STROBE guidelines.^47^

## Results

### Study participants

**Figure 2:**
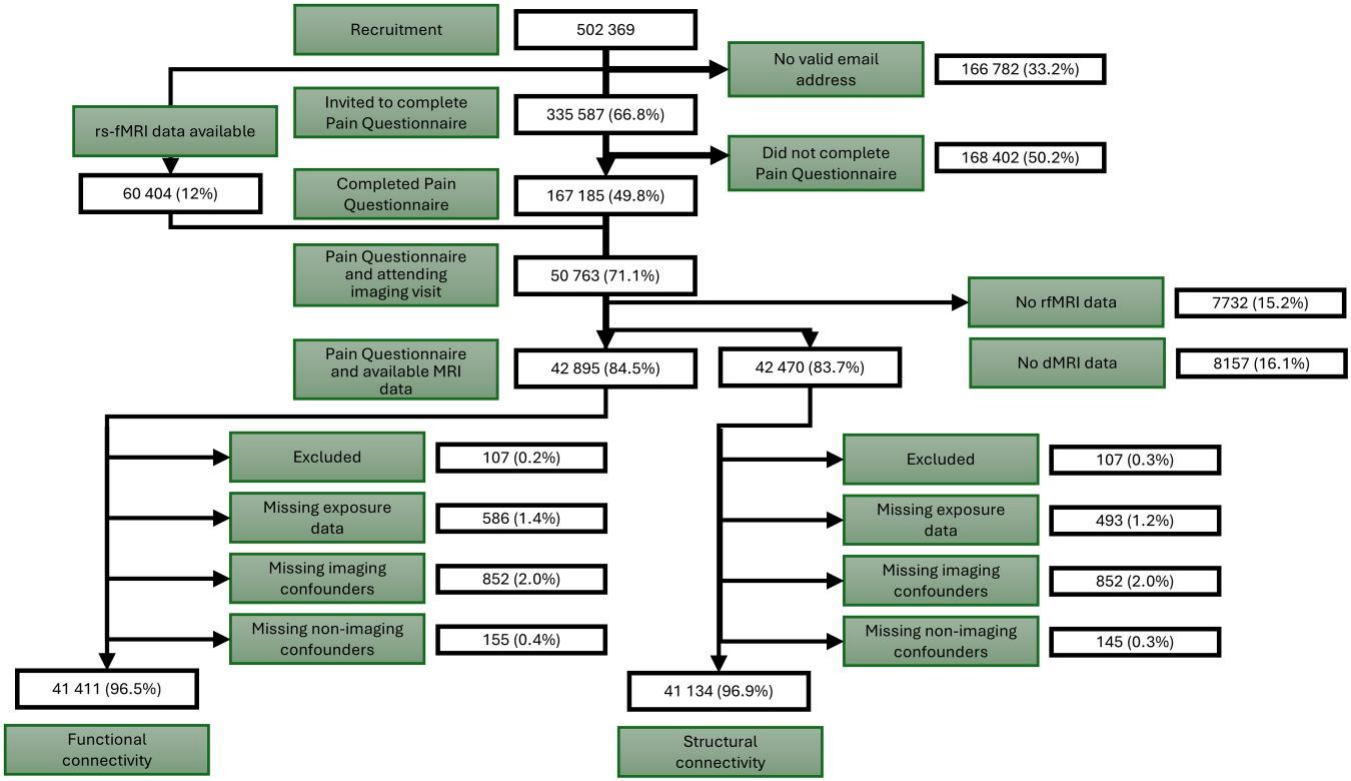
**Study flow diagram for UK Biobank participants included in analyses of DPMS connectivity and nociplastic pain severity**. Functional connectivity (rfMRI) data were available for 41 411 participants scanned between May 2014 and June 2023, while structural connectivity (dMRI) data were extracted for a subset of 41 134 participants scanned between August 2014 and June 2023. The ‘Excluded’ group comprises participants with major neurological condition at baseline who were excluded from this analysis. dMRI = diffusion-weighted MRI; DPMS = descending pain modulatory system; rfMRI = resting-state functional MRI.

Of the 167 185 UK Biobank participants who completed the 2019 pain questionnaire, 50 763 attended the neuroimaging assessment between May 2014 and June 2023 and functional connectivity (rfMRI) data were available for 42 895 (84.5%) participants, while structural connectivity (dMRI) data were extracted for a subset of 42 470 (83.7%) participants. Functional and structural connectivity metrics were successfully extracted for 99.9% and 98.6% of participants with available fMRI and dMRI imaging data, respectively. A small proportion of participants (<5%) were subsequently excluded due to missing pain questionnaire responses or incomplete confounder data (e.g. head size, scan date, head motion), leaving a sample size of 41 411 for the functional connectivity, and 41 134 for the structural connectivity, analyses. Figure 2 summarizes the population flow diagram for this study.

### Baseline characteristics

Baseline characteristics of participants included in the functional connectivity analysis are detailed in Table 2. The mean age was 64.7 years (SD 7.61), 54% were female. Most participants were retired (59%) and predominantly of white ethnicity (97.4%). Most (71%) reported weekly or daily alcohol consumption, while only 3.1% were current smokers. The mean BMI was 26.3 kg/m^2^. Participants with chronic pain (53% of the cohort) showed higher FMI scores compared to those without chronic pain, with mean WPI scores of 2.18 versus 0.28 and mean SSS scores of 2.94 versus 1.69. Sociodemographic and lifestyle factors were largely similar between individuals with and without chronic pain, although a slightly higher proportion of chronic pain participants were female (58% versus 50%).

**Table 2:** Baseline characteristics of participants included in analysis of DPMS connectivity and nociplastic pain severity

	Total	No chronic pain	Chronic pain
	(*N* = 41 411)	(*n* = 19 272)	(*n* = 22 139)
Female, *n* (%)	22 375 (54%)	9616 (50%)	12 759 (58%)
Age, mean (SD) years	64.7 (7.61)	64.6 (7.63)	64.8 (7.59)
Townsend Deprivation Index, mean (SD)	−1.90 (2.73)	−1.96 (2.70)	−1.86 (2.75)
Married/partner, *n* (%)	30 866 (75%)	14 458 (75%)	16 408 (74%)
Employment status			
Employed	15 087 (36%)	7290 (38%)	7797 (35%)
Retired	24 580 (59%)	11 288 (59%)	13 292 (60%)
Unemployed/other	1509 (4%)	590 (3%)	919 (4%)
White ethnicity, *n* (%)	42 516 (97.4%)	18 790 (97.5%)	21 541 (97.3%)
University degree, *n* (%)	21 327 (51.5%)	10 445 (54.2%)	10 892 (50%)
Current tobacco use, *n* (%)	1265 (3.1%)	561 (1.7%)	704 (3.2%)
Alcohol use			
Never	2695 (7%)	1171 (6%)	1524 (7%)
Rarely	9010 (22%)	3886 (20%)	5124 (23%)
Weekly	22 493 (54%)	10 773 (56%)	11 720 (53%)
Daily	6971 (17%)	3334 (17%)	3637 (16%)
Body mass index, kg/m^2^, mean (SD)	26.3 (4.38)	25.9 (4.08)	26.8 (4.58)
Fibromyalgia Index (0–31), mean (SD)	3.66 (3.55)	1.97 (1.93)	5.13 (3.96)
Widespread Pain Index (0–19), mean (SD)	1.30 (2.00)	0.279 (0.752)	2.18 (2.30)
Symptom Severity Scale (0–12), mean (SD)	2.36 (2.11)	1.69 (1.64)	2.94 (2.30)

Sociodemographic, lifestyle, pain-related, and cognitive performance metrics are summarized for participants (*N* = 41 411). Results are presented as percentages, means with standard deviations (SD), or medians with ranges, as appropriate. Higher values of Townsend Deprivation Index indicate greater social deprivation. Nociplastic pain assessed using the Fibromyalgia Index, with higher scores indicating more severe nociplastic pain. The Fibromyalgia Index is the sum of the Widespread Pain Index and Symptom Severity Scale. DPMS = descending pain modulatory system.

### Objective 1: associations between DPMS connectivity and FMI score

Omnibus F-tests demonstrated that both functional (*P =* 0.0002) and structural (*P* = 0.003) DPMS connectivity significantly explained variance in FMI scores in the full sample (Table 3). Structural connectivity effects were primarily driven by participants with chronic pain (*P =* 0.0001), whereas functional connectivity was significantly associated with FMI in both chronic pain (*P* = 0.021) and no chronic pain groups (*P =* 0.016).

**Table 3:** Differential contribution of functional and structural DPMS connectivity to nociplastic pain severity

Group	*Df*	*F*	*P*	*P* LRT (CP versus NP)
**Functional connectivity**				
All	6	4.410	0.0002	–
Chronic pain	6	2.487	0.021	–
No chronic pain	6	2.600	0.016	0.466
**Structural connectivity**				
All	6	3.347	0.003	–
Chronic pain	6	4.565	0.0001	–
No chronic pain	6	0.749	0.610	0.0015

Omnibus F-tests comparing full and reduced linear regression models to evaluate whether sets of six functional or structural DPMS connectivity edges collectively explain variance in nociplastic pain severity (Fibromyalgia Index), stratified by chronic pain status. *Df* = degrees of freedom; *F* = F-statistic; ‘*P* LRT (CP versus NP)’ refers to the *P*-value from the likelihood ratio test (LRT) comparing constrained versus unconstrained structural equation model across chronic pain (CP) and no pain (NP) groups. DPMS = descending pain modulatory system.

#### Objective 1a: associations between functional connectivity in the DPMS and FMI score

**Figure 3:**
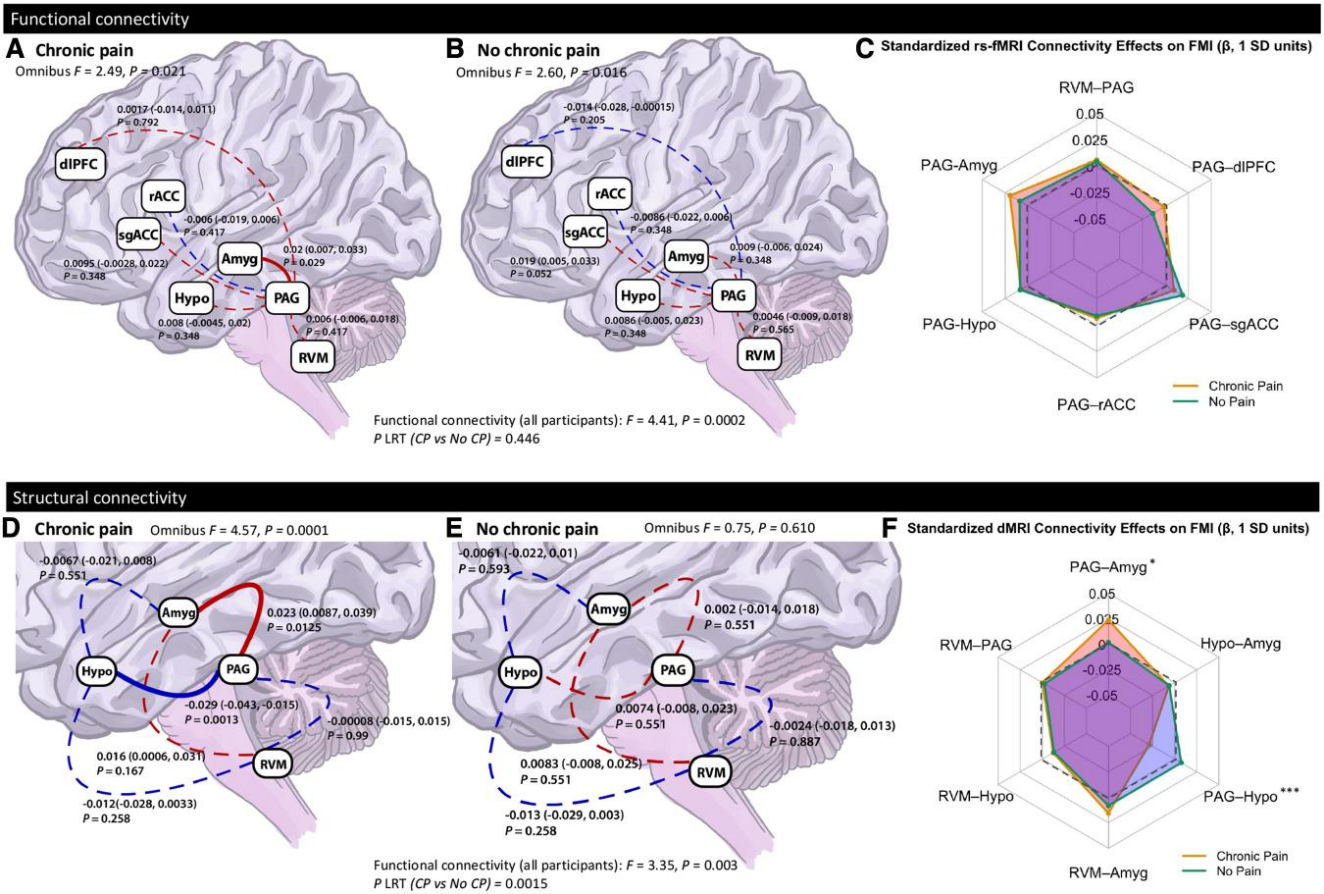
**Fibromyalgia Index score is associated with functional and structural connectivity in the descending pain modulation system in adults with chronic pain**. Multi-group structural equation modelling (SEM) assessed the relationship between descending pain modulatory system (DPMS) connectivity and Fibromyalgia Index (FMI) scores in participants with and without chronic pain. All connectivity *P*-values were false discovery rate-corrected across DPMS edges within each model. (**A** and **B**) Functional connectivity. Red lines indicate positive associations, and blue lines indicate negative associations; solid lines represent statistically significant paths (*P* < 0.05), and dashed lines are non-significant. Standardized coefficients and bootstrapped confidence intervals and corrected *P*-values presented. (**C**) Radar chart summarizing standardized path estimates (β, in SD units) from the SEM model linking resting-state functional MRI (rfMRI) connectivity to FMI. Each spoke represents a unique connection; orange = Chronic Pain group, green = No Chronic Pain group. The dashed grey polygon denotes a reference ring at β = 0. (**D** and **E**) Structural connectivity. Red lines indicate positive associations, and blue lines indicate negative associations; solid lines represent statistically significant paths (*P* < 0.05), and dashed lines are non-significant. Standardized coefficients and bootstrapped confidence intervals and *P*-values presented. (**F**) Radar chart summarizing standardized β estimates from the diffusion-weighted MRI SEM model, as in **C**. The chart visualizes direction and magnitude of connectivity–FMI associations across groups. Each spoke represents a unique connection; orange = Chronic Pain group, green = No Chronic Pain group. The dashed grey polygon denotes a reference ring at β = 0. Amyg = amygdala; dlPFC = dorsolateral prefrontal cortex; Hypo = hypothalamus; PAG = periaqueductal grey; rACC = rostral anterior cingulate cortex; RVM = rostral ventromedial medulla; SD = standard deviation; sgACC = subgenual anterior cingulate cortex.

Multi-group SEM revealed no significant difference in the association between functional connectivity of the DPMS and FMI scores by chronic pain status (LRT *P* = 0.446; Fig. 3). PAG-amygdala connectivity was associated with FMI in the chronic pain group (β = 0.02, 95% CI: 0.007 to 0.033, *P* = 0.029; Fig. 3A). A nominal group difference for this edge was identified via the Wald test but was not statistically significant after correction for multiple comparisons (*P* = 0.217). A comparison of associations in the chronic pain versus no chronic pain group are displayed in Fig. 3C. Detailed results are presented in Supplementary Tables 2 and 3.

#### Objective 1b: structural connectivity in DPMS is associated with FMI in chronic pain

Structural connectivity between DPMS regions showed significant differences in their relationship with FMI scores by chronic pain status (LRT *P* = 0.0015; Fig. 3). In participants with chronic pain, the PAG-amygdala (β = 0.023, 95% CI: 0.0087 to 0.039, *P* = 0.0125; Fig. 3D) and PAG-hypothalamus (β = −0.029, 95% CI: −0.043 to −0.015, *P* = 0.0013; Fig. 3D) edges were significantly associated with FMI scores. No significant associations were observed in those without chronic pain (Fig. 3E). Wald tests confirmed significant group differences for PAG-amygdala (*P* = 0.028) and PAG-hypothalamus edges (*P* = 0.0007), suggesting that structural DPMS connectivity is differentially associated with FMI depending on chronic pain status (Fig. 3F). Full results are presented in Supplementary Tables 4 and 5.

### Objective 2: mediation with SPACE and WPI

**Figure 4:**
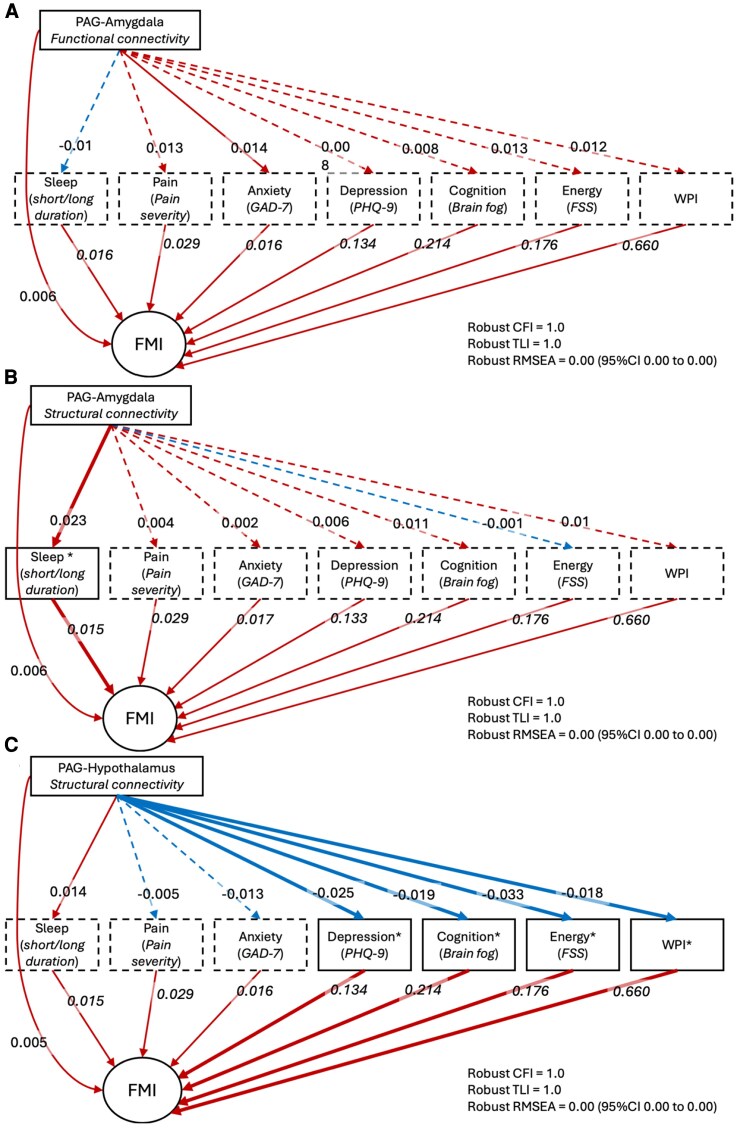
**Mediation models evaluating whether SPACE symptoms and widespread pain explain associations between DPMS connectivity and nociplastic pain severity**. Structural equation models were used to assess whether symptoms from the SPACE cluster—sleep disturbance, pain, affect (depression/anxiety), cognitive symptoms, and low energy—along with Widespread Pain Index (WPI), mediate associations between connectivity in the descending pain modulatory system (DPMS) and Fibromyalgia Index (FMI), in adults with chronic pain. Each panel represents a separate mediation model. All connectivity *P*-values were false discovery rate-corrected across DPMS edges within each model. (**A**) PAG-amygdala functional connectivity; (**B**) PAG-amygdala structural connectivity; and (**C**) PAG-hypothalamus structural connectivity. Arrows represent the direction of associations: from each brain metric (*top*) to mediators (*middle*), and from mediators to FMI. The direct path from the brain metric to FMI is also included. Standardized path coefficients and corresponding bootstrapped 95% confidence intervals and *P*-values presented. Red arrows represent positive associations, while blue arrows represent negative associations. Solid lines denote statistically significant paths (*P* < 0.05), while dashed lines indicate non-significant paths. All models demonstrated excellent fit, with Comparative Fit Index (CFI) and Tucker-Lewis Index (TLI) values of 1.0, and root mean square error of approximation (RMSEA) of 0.00. FSS = Fatigue Severity Scale; GAD-7 = Generalized Anxiety Disorder-7; PAG = periaqueductal grey; PHQ-9 = Patient Health Questionnaire-9 (depression). Refer also to Table 4.

Mediation models evaluated whether the relationship between DPMS connectivity and FMI was explained by common features of nociplastic pain, including SPACE symptoms and widespread pain (Fig. 4 and Table 4). There was no evidence of multicollinearity between the mediators (VIF < 5 for all; Supplementary Table 6). Model fit was excellent for all models. Among participants with chronic pain, PAG-amygdala functional connectivity showed a significant total indirect effect on FMI (β = 0.013, 95% CI: 0.0013 to 0.026, *P* = 0.030; Fig. 4A and Table 4), largely driven by paths through fatigue and widespread pain, although individual mediators did not reach significance (Table 4). The direct effect remained significant (β = 0.006, 95% CI 0.002 to 0.032; *P* = 0.003), suggesting partial mediation.

**Table 4:** Standardized path coefficients for mediation models assessing the role of SPACE symptoms and widespread pain in associations between DPMS connectivity and nociplastic pain severity

Path	β (95% CI)	*P* (uncorrected)	*P* (corrected)
**PAG-amygdala functional connectivity**			
Indirect pathways			
Sleep	−0.00016 (−0.00042 to 0.00005)	0.125	0.292
Pain severity	0.00039 (−0.00001 to 0.00078)	0.058	0.820
Depression	0.00105 (−0.00069 to 0.00279)	0.236	0.599
Anxiety	0.00023 (−0.00002 to 0.00048)	0.068	0.922
Brain fog	0.00166 (−0.00114 to 0.00445)	0.246	0.292
Fatigue	0.00225 (−0.00006 to 0.00455)	0.056	0.922
Widespread pain	0.00802 (−0.00056 to 0.01661)	0.067	0.105
Total indirect	**0.01343** (0.00128 to 0.02558)	0.030	–
Direct	**0.00604** (0.00211 to 0.00996)	0.003	–
Total	**0.01947** (0.00662 to 0.03232)	0.003	–
**PAG-amygdala structural connectivity**			
Indirect pathways			
Sleep	**0.00036** (0.00019 to 0.00084)	0.0017	0.012
Pain severity	0.00011 (−0.00027 to 0.00050)	0.560	0.784
Depression	0.00078 (−0.00082 to 0.00237)	0.342	0.599
Anxiety	0.00003 (−0.00029 to 0.00038)	0.798	0.922
Brain fog	0.00240 (−0.00043 to 0.00522)	0.097	0.340
Fatigue	−0.00011 (−0.00235 to 0.00212)	0.922	0.922
Widespread pain	0.00639 (−0.00256 to 0.01533)	0.162	0.378
Total indirect	0.00995 (−0.00258 to 0.02249)	0.120	–
Direct	**0.00640** (0.00231 to 0.01048)	0.002	–
Total	**0.01635** (0.00298 to 0.02972)	0.017	–
**PAG-hypothalamus structural connectivity**			
Indirect pathways			
Sleep	0.00022 (0.00000 to 0.00058)	0.053	0.074
Pain severity	−0.00015 (−0.00057 to 0.00026)	0.473	0.473
Depression	**−0.00335** (−0.00506 to −0.00163)	<0.001	<0.001
Anxiety	−0.00022 (−0.00048 to 0.00003)	0.088	0.102
Brain fog	**−0.00399** (−0.00688 to −0.00109)	0.007	0.016
Fatigue	**−0.00575** (−0.00811 to −0.00339)	<0.001	<0.001
Widespread pain	**−0.01186** (−0.02087 to −0.00285)	0.010	0.017
Total indirect	**−0.02509** (−0.03769 to −0.01249)	<0.001	–
Direct	**0.00520** (0.00123 to 0.00918)	0.010	–
Total	**−0.01989** (−0.03315 to −0.00662)	0.003	–

Indirect, direct and total effects for each mediation model are presented, with bootstrapped 95% confidence intervals (CI) and both uncorrected and false discovery rate-corrected *P*-values. Results are reported separately for models examining (*top*) PAG-amygdala functional connectivity; (*middle*) PAG-amygdala structural connectivity; and (*bottom*) PAG-hypothalamus structural connectivity (refer also to Fig. 4). DPMS = descending pain modulatory system; PAG = periaqueductal grey; SPACE = sleep disturbance, pain, affect, cognitive problems and low energy.

In contrast, PAG-amygdala structural connectivity was associated with a significant indirect effect via sleep duration (β = 0.0004, 95% CI: 0.0002 to 0.0008, *P* = 0.002; Fig. 4B and Table 4), while the direct effect remained robust (β = 0.006, 95% CI: 0.002 to 0.01; *P* = 0.002; Fig. 4B and Table 4). This suggests partial mediation by sleep in the pathway linking PAG-amygdala structural connectivity to nociplastic symptom severity.

Structural connectivity between the PAG and hypothalamus showed a significant negative indirect effect on FMI (β = −0.025, 95% CI: −0.038 to −0.012, *P* < 0.001; Fig. 4C and Table 4), primarily via depression, brain fog, fatigue, and widespread pain. A smaller positive direct effect was also observed (β = 0.005, 95% CI 0.001 to 0.009; *P* = 0.010; Fig. 4C and Table 4), indicating opposing indirect and direct contributions.

As a sensitivity analysis, we fitted a model in which SPACE symptoms were modelled as a single latent factor to account for their shared variance (Supplementary Fig. 2). This approach yielded similar overall patterns of association. For PAG-hypothalamus structural connectivity, there was a significant indirect effect on FMI mediated via the SPACE latent factor (β = −0.019, 95% CI: −0.032 to −0.012; *P* < 0.001), consistent with partial mediation. In contrast, indirect effects via the latent SPACE factor were not statistically significant for PAG-amygdala functional connectivity (β = 0.008, 95% CI: 0.0002 to 0.016; *P* = 0.042 uncorrected, *P* = 0.067 FDR-corrected) or structural connectivity (β = 0.004, 95% CI: −0.004 to 0.017; *P* = 0.296), suggesting limited additional explanatory power of the latent model for those edges.

Together, these results suggest that structural DPMS connectivity is more strongly associated with nociplastic symptom burden than functional connectivity. These associations may be partially mediated by common symptoms of nociplastic pain, including widespread pain and somatic symptoms such as fatigue and brain fog. The FMI score reflects these complex pathways, and is sensitive to both the pain and non-pain dimensions apparent in this group.

### Objective 3: other pain phenotypes are not associated with DPMS connectivity

No significant associations were found between DN4 or pain intensity (NRS) scores and DPMS functional or structural connectivity (Supplementary Tables 7–10).

### Exploratory age-stratified analysis

For functional connectivity, a significant overall group difference in FMI scores was observed across age bands (LRT *P* = 0.023). This was primarily driven by age-related differences in PAG-dlPFC connectivity, which showed a significant interaction with age group [Wald χ^2^(5) = 22.6, *P* = 0.002]. Specifically, a positive association between DPMS functional connectivity and FMI was observed in younger adults (40–45 years: β = 0.043, 95% CI 0.013 to 0.073; *P* = 0.005), while an inverse association emerged in older adults (66–70 years: β = −0.053, 95% CI −0.081 to −0.025; *P* < 0.001) (Supplementary Tables 11 and 12). In contrast, structural connectivity effects on FMI did not significantly vary across age groups (LRT *P* = 0.451), and no individual structural edge showed a significant age interaction after FDR correction (all *P*_corr_ > 0.05; Supplementary Tables 13 and 14).

## Discussion

### Summary of key findings

In this study, we demonstrate that the FMI is associated with altered structural and functional connectivity within the DPMS in a large, population-based sample of individuals with chronic pain. These findings support the FMI as a marker of nociplastic pain severity at the population level.

The strongest associations were observed for FMI and structural connectivity between the PAG with the amygdala and hypothalamus, highlighting the role of the lower DPMS in nociplastic pain. These circuits are well-established in integrating affective, autonomic, and sensory dimensions of pain. The PAG-amygdala pathway was linked with sleep disturbance, while PAG-hypothalamus connectivity was associated with mood, fatigue, and widespread pain. These distinct patterns suggests that specific DPMS subcircuits may support different symptom domains of nociplastic pain.

Importantly, these associations were observed only in individuals with chronic pain, reinforcing the specificity of DPMS alterations to nociplastic mechanisms rather than to general affective or somatic symptoms observed in the absence of chronic pain. The lack of association with other pain measures, such as the DN4 or pain intensity, further supports the relevance of the FMI to nociplastic pain and its underlying central mechanisms.

Exploratory age-stratified analyses suggested that associations between DPMS functional connectivity and nociplastic symptom severity may vary across the adult lifespan. Notably, PAG-dlPFC connectivity showed a positive association with FMI scores in younger adults, but an inverse association in older adults, suggesting possible age-related shifts in the functional role of this pathway. In contrast, structural connectivity associations with FMI appeared stable across age groups. However, the age range in UK Biobank is relatively narrow, and studies in cohorts with a wider age distribution, particularly children and young adults, would be valuable to further explore developmental trajectories in nociplastic pain.

Structural alterations in the DPMS were more strongly associated with FMI scores than functional connectivity. This may reflect the cumulative impact of chronic nociplastic symptoms on white matter pathways, which provide the anatomical scaffold for functional brain networks.^48^ Structural architecture constrains the range of possible functional interactions^49^ and regions with direct anatomical connections are more likely to exhibit functional connectivity during both rest and task conditions.^50^ Although functional coupling can occur via indirect or polysynaptic pathways, structurally connected regions, such as those within the DPMS (e.g. PAG, RVM, ACC),^27^ exhibit more consistent co-activation, particularly during evoked pain.^49^ In chronic pain, structural connectivity may also reflect vulnerability or resilience: white matter integrity between the nucleus accumbens and medial prefrontal cortex has been shown to predict pain chronification,^51^ and pathways linking the DLPFC, thalamus, PAG and ACC are associated with analgesic responses to tDCS and placebo.^52,53^ Importantly, the relationship between structure and function is bidirectional: repeated functional co-activation can strengthen white matter tracts,^54^ while age-related structural decline can weaken functional connectivity.^55^ Although functional reorganisation may compensate for structural disruption, such compensation may only emerge under challenge, such as evoked pain. This dynamic interplay may explain why structural connectivity showed stronger associations with FMI scores than resting-state measures in this study.

### Existing literature

#### Fibromyalgia Index

As highlighted in a recent systematic review, no single clinical tool reliably distinguishes nociplastic from nociceptive or neuropathic pain, and most existing methods are insufficiently validated for this purpose.^56^ Despite these limitations, fibromyalgia remains the prototypical condition for studying nociplastic pain, and the FMI offers a practical, if imperfect, index of this phenotype. Although the 2019 IASP clinical grading criteria for nociplastic pain have been proposed, they require clinical examination for pain hypersensitivity and have not been validated in large-scale studies or related to underlying neurobiological changes.^57^ In contrast, the FMI is readily available in many population-based datasets, such as UK Biobank, and captures core features of nociplastic pain, including widespread pain and somatic symptom burden, on a continuous scale. This allows for the investigation of nociplastic pain as a continuum, rather than a binary state.^58^

Previous studies have used the FMI to examine pain mechanisms across a range of conditions, including rheumatoid arthritis,^59^ ankylosing spondylitis,^60^ Sjögren's syndrome,^61^ systemic lupus erythematosus,^62^ vasculitis^63^ and endometriosis.^64^ FMI scores have been shown to predict postoperative pain outcomes following orthopaedic surgery^14,65^ and hysterectomy.^66^ In neuroimaging studies, increased connectivity between the default mode network and the posterior insula has been correlated with FMI scores in rheumatoid arthritis.^59^ supporting a centralized pain phenotype. However, notably, these studies often used task-based functional MRI paradigms, in contrast to our resting-state and diffusion-based analysis. Nevertheless, the convergence of FMI with both clinical and neuroimaging correlates across diverse pain conditions reinforces its utility as a population-level marker of nociplastic pain.

#### Descending pain modulation system

The DPMS serves a dual function in pain modulation, capable of both inhibiting and facilitating nociceptive processing depending on the context.^6^ The PAG plays a pivotal role as a hub integrating top-down signals from subcortical and cortical areas, such as the dlPFC and ACC, with bottom-up nociceptive input from the spinal cord.^10^ Its role extends beyond pain modulation, encompassing autonomic responses and defensive behaviours.^27,67-69^ Evidence from neuroimaging studies suggests that this balance between facilitation and inhibition can become disrupted in chronic pain, leading to a pro-nociceptive state characterized by hypersensitivity and reduced pain inhibition.^6,10^

Structural and functional changes in PAG connectivity are well-documented in fibromyalgia and other chronic pain conditions.^70-73^ Tractography studies have shown that the PAG is connected to key DPMS regions, including the hypothalamus, amygdala and PFC.^27^ In healthy adults, an RVM-PAG-ACC resting state network supports the functional integration of the DPMS.^7^

We found that PAG-amygdala connectivity was associated with nociplastic pain severity at a population-level. This aligns with evidence supporting the amygdala's role in the emotional and cognitive modulation of pain, serving as a relay between the PAG-RVM axis and higher cortical regions.^5,74^ The amygdala is implicated in the fear response and anticipatory aspects of pain and may mediate the dysregulated affective control observed in fibromyalgia. Previous studies have reported reduced amygdala volume^75^ and altered functional connectivity with other DPMS nodes, such as the ACC.^76^ Our results are consistent with the known contributions of the amygdala to stress and arousal states, which exacerbate pain perception, supporting the notion that nociplastic pain is influenced by a complex interplay of biological, psychological, and social factors. This aligns with pain vulnerability models, which suggest that altered neural circuits may predispose individuals to chronic pain by amplifying the impact of psychosocial stressors.^77^

The PAG-hypothalamus circuit integrates nociceptive, affective and autonomic inputs and is essential for descending pain inhibition.^78,79^ Disruption of orexin-mediated analgesic signalling from the hypothalamus to PAG, as shown in rodent models, may impair endogenous pain control and promote sensitization.^80^ These findings support our observed associations between reduced PAG-hypothalamus connectivity and greater nociplastic symptom severity and highlight this pathway's potential role in linking pain with broader homeostatic and emotional dysregulation.

Latent sensitization provides a framework for understanding the transition from acute to chronic pain in the context of altered DPMS function, where there is a compensatory increase in descending inhibitory tone following injury, masking ongoing sensitization. When this mechanism fails, unmasked hyperalgesia emerges, which may contribute to the persistence of chronic pain.^81^ The observed changes in DPMS connectivity may reflect a similar exhaustion of compensatory inhibitory processes, resulting in the pro-nociceptive states associated with nociplastic pain.^82^

Our study addresses an important gap in the literature by linking DPMS connectivity to a continuous measure of nociplastic pain severity in a population-based sample. Moreover, our mediation findings suggest that connectivity changes within the DPMS may underlie key features of nociplastic pain, including widespread pain, fatigue, mood disturbance, and sleep dysfunction.^4^ The alignment of DPMS connectivity with these clinical features supports the validity of the FMI and enhances our understanding of the neural basis of nociplastic pain.

### Strengths and weaknesses

This is the first study to evaluate the neurobiological correlates of the FMI in a large, population-based cohort. We used both structural and functional MRI, allowing for a multimodal assessment of the DPMS. All models were adjusted for a comprehensive set of imaging and non-imaging confounders.

However, several limitations must be acknowledged. First, this is a cross-sectional study, and the direction of association between connectivity measures and pain cannot be deduced. This is further limited by the nature of data collection in UK Biobank, where the FMI was collected separately from the neuroimaging visit. Although the large sample size is a strength, it may allow the detection of statistically significant small effects of little clinical relevance. However, these are beneficial to improving our understanding of the underlying neurobiology of nociplastic pain and guiding future targeted studies in clinical populations. Furthermore, the neuroimaging sequences used in UK Biobank were not optimized for brainstem structures, potentially introducing noise into PAG and RVM measurements. Although resting-state functional connectivity can predict behaviour and activations during task fMRI,^83-85^ it may be relatively insensitive to changes in the DPMS. Additionally, the reliance on indirect measures of DPMS function, such as resting-state functional connectivity, limits the ability to distinguish between facilitation and inhibition. It is important to note that the interpretation of blood oxygen level-dependent activity in PAG-amygdala connectivity as either pro-nociceptive or anti-nociceptive remains challenging due to the limitations of resting-state neuroimaging in distinguishing between facilitation and inhibition within the DPMS. Future studies employing task-based fMRI paradigms or pharmacological manipulations targeting the PAG-amygdala circuit are needed to disentangle these mechanisms and clarify their functional implications in nociplastic pain. This study is also limited by the selection of both neuroimaging and behavioural variables, and by the definition of the ROI masks. Replication of these findings in independent datasets using independent masks would reinforce the observed associations. Quantitative sensory testing measures evaluating DPMS function, such as conditioned pain modulation and temporal summation, were not performed in the UK Biobank, although findings from these techniques have not been very reproducible.^86^ Another limitation is the absence of a gold-standard measure for nociplastic pain. While the FMI captures many core features, it does not assess evoked pain hypersensitivity or sensory amplification, which are central to the construct. Selection bias in UK Biobank must also be acknowledged, as participants tend to be healthier and more educated than the general population.

## Conclusion

In conclusion, we demonstrate that the FMI is associated with altered structural and functional connectivity in the DPMS in a large population-based study, particularly in circuits involving the PAG, amygdala, and hypothalamus. These connectivity patterns are also linked to hallmark features of nociplastic pain, including fatigue, mood disturbance, sleep dysfunction, and widespread pain. Our findings provide support for the FMI as a marker of nociplastic pain severity in the general population with chronic pain and highlight the role of the lower DPMS in its neurobiological underpinnings. These insights can guide future research and contribute to the development of mechanism-based approaches to the stratification and treatment of nociplastic pain.

## Supplementary Material

awaf307_Supplementary_Data

## Data Availability

Data is available upon application to the UK Biobank, https://www.ukbiobank.ac.uk/enable-your-research/apply-for-access. All analysis code will be made publicly available upon publication on an online repository.
