# Supplementary material for: Brain signatures of nociplastic pain: Fibromyalgia Index and descending modulation at population level
Source: Brain. 2025 Aug 17;149(4):1365–80. doi: 10.1093/brain/awaf307 (PMC7618718; doi:10.1093/brain/awaf307)
Supplement: awaf307_Supplementary_Data [file awaf307_supplementary_data.pdf]

Supplementary materials

Supplementary Table 1. Field IDs for variables from UK Biobank used in the analysis.

Instance 2, imaging visit. PQ, 2019 pain questionnaire.

Variable	Field ID	Instance	Comments
Age at imaging	21003	2	
Sex	31	0	
Ethnicity	21000	2	Binarised to white or non-white. Imputed with instance 0 if missing at 2
Townsend Deprivation Index	22189	0	
Education	6138	2	Binarised to University Degree and No Degree. Instance 0 or 10722 used if 6138 missing.
Employment status	6142	2	Binarised to employed or not employed
Smoking	20116	2	Binarised to current smoking or not current smoker
Alcohol use	1558	2	Binarised to current alcohol use or no current alcohol use
BMI	21001	2	Instance 0 or 23104 used if 21001 missing
Sleep duration	1160	2	
Insomnia symptoms	1200	2	
Chronic pain at imaging		2	Answered "Yes" to any of 3799, 3403, 3571, 3741, 3414, 3773 or 2956.
Headaches for 3+ months	3799	2	
Facial pain for 3+ months	4067	2	
Neck/shoulder pain for 3+ months	3403	2	
Back pain for 3+ months	3571	2	
Stomach/Abdominal pain for 3+ months	3741	2	
Hip pain for 3+ months	3414	2	
Knee pain for 3+ months	3773	2	
General pain for 3+ months	2956	2	
Number of pain sites at imaging		2	Sum of 3799, 3403, 3571, 3741, 3414 and 3773.
Fibromyalgia Index		PQ	Sum of Widespread Pain Index and Symptom Severity Scale
Widespread Pain Index	120039	PQ	
Fatigue (SSS)	120040	PQ	
Unrefreshing sleep (SSS)	120041	PQ	
Cognitive difficulties (SSS)	120042	PQ	
Abdominal pain (SSS)	120043	PQ	
Depression (SSS)	120044	PQ	

**Variable**	**Field ID**	**Instance**	**Comments**
Headache (SSS)	120045	PQ	
Symptom severity Scale		PQ	sum of 120039-120045
Depression (PHQ-9)	120104-120112	PQ	Sum of 120104-120112
Fatigue Severity Scale (FSS)	120119-120127	PQ	Sum of 120119-120127, not answered if 120018 is "No" and 120040 is "No problem". In that case the variable was set at the minimum value (i.e. 9)
Pain severity (NRS)	120022 & 12086	PQ	
Neuropathic Pain (DN4)	120046-120052	PQ	Sum of 120046-120052. Only answered participant reported chronic pain in a body site (not answered if participant reported chronic pain "all over the body"
Date of Pain Questionnaire	120128	PQ	
Age at pain assessment			Derived from field 31 and date of PQ
Follow-up time from imaging to pain questionnaire			The difference between baseline date (53) and pain questionnaire date (120128)
Anxiety (GAD-7)	29058-29064	MWBQ	sum of 29058-29064
Exclusion	20002	2	If contained codes: 1262 (dementia), 1263 (Parkinson's), 1289 (psychosis), or 1291 (bipolar disorder)
Imaging confounds			
scan_date	53	2	scan date
Site	54	2	imaging site
Age	21003	2	age
Sex	31	2	sex
HeadSize	25000	2	head size scaling
TablePos_Table	25759	2	scanner table position
TablePos_COG_X	25756	2	Head centre of gravity in scanner coordinates (X)
TablePos_COG_Y	25757	2	Head centre of gravity in scanner coordinates (Y)
TablePos_COG_Z	25758	2	Head centre of gravity in scanner coordinates (Z)
HeadMotion_mean_rfMRI_rel	25741	2	head motion in resting fMRI (mean relative motion as calculated by FEAT)

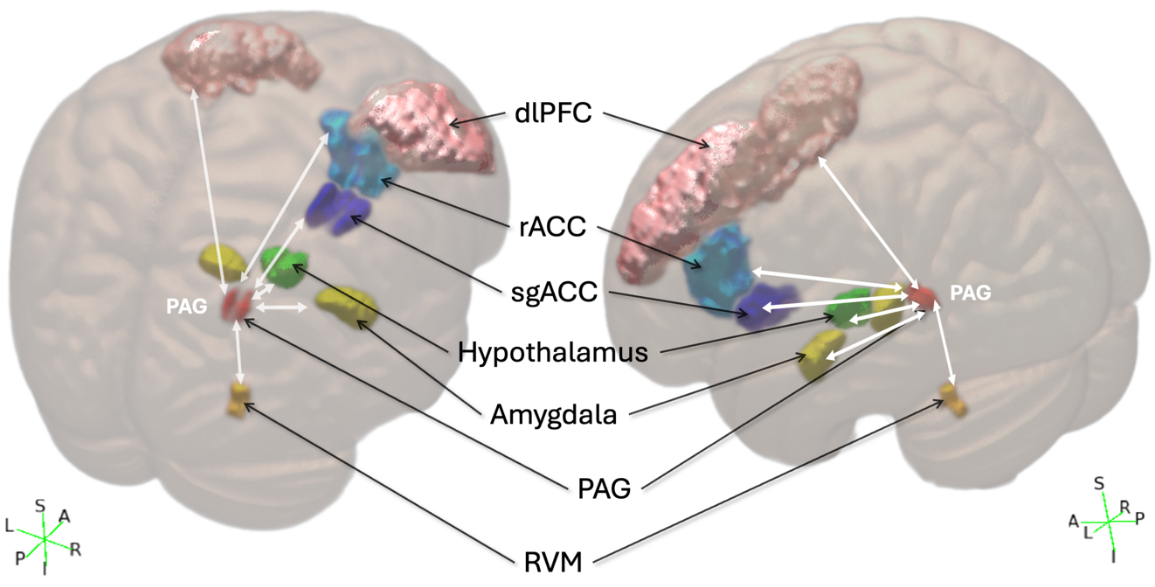

Supplementary Figure 1. Three-dimensional (3D) representation of the region of interest masks of DPMS evaluated in this study.

Masks are overlayed on 3D MNI152 standard space brain. DPMS, descending pain modulation system. RVM, rostral ventromedial medulla. PAG, periaqueductal grey. rACC, rostral anterior cingulate cortex. sgACC, subgenual anterior cingulate cortex. dlPFC, dorsolateral prefrontal cortex. S, superior. I, inferior. A, anterior. P, posterior. L, left. R, right.

Supplementary Table 2. Standardized estimates of associations between functional connectivity within the descending pain modulatory system (DPMS) and FMI scores, stratified by chronic pain status.
Standardized β coefficients, 95% confidence intervals (CI), standard errors (SE), Z-values, and P-values are reported for each connectivity path from the unconstrained multi-group SEM. Results are shown separately for participants with and without chronic pain. False discovery rate (FDR) correction was applied across all 12 connectivity paths (6 per group) using the Benjamini–Hochberg procedure (Pcorr). No overall group difference in connectivity effects was observed (likelihood ratio test P = 0.446).

	**β**	**95%CI lower**	**95%CI upper**	**SE**	**Z**	**P**	**P_{corr}**
**Chronic Pain**							
RVM-PAG	0.006	-0.007	0.019	0.006	0.939	0.348	0.417
PAG-amygdala	0.020	0.007	0.032	0.006	3.030	0.002	0.029
PAG-hypothalamus	0.008	-0.005	0.020	0.006	1.195	0.232	0.348
PAG-rACC	-0.006	-0.019	0.007	0.007	-0.959	0.337	0.417
PAG-sgACC	0.009	-0.003	0.022	0.007	1.459	0.145	0.348
PAG-dlPFC	-0.002	-0.014	0.011	0.006	-0.264	0.792	0.792
**No Chronic Pain**							
RVM-PAG	0.005	-0.009	0.018	0.007	0.646	0.518	0.565
PAG-amygdala	0.009	-0.005	0.023	0.007	1.264	0.206	0.348
PAG-hypothalamus	0.009	-0.005	0.023	0.007	1.219	0.223	0.348
PAG-rACC	-0.009	-0.023	0.005	0.007	-1.196	0.232	0.348
PAG-sgACC	0.019	0.005	0.033	0.007	2.625	0.009	0.052
PAG-dlPFC	-0.014	-0.028	0.000	0.007	-1.949	0.051	0.205

Supplementary Table 3. Group differences in functional connectivity-FMI associations assessed via Wald tests.
Wald χ^2 statistics, degrees of freedom, and P-values for each connectivity edge between the periaqueductal grey (PAG) and target regions within the descending pain modulatory system (DPMS). False discovery rate (FDR) correction was applied across all 6 connectivity paths using the Benjamini–Hochberg procedure (Pcorr).

**Edge**	**ChiSq**	**Df**	**P**	**P_{corr}**
PAG-amygdala	4.387	1	0.036	0.217
RVM-PAG	0.275	1	0.600	0.931
PAG-hypothalamus	0.230	1	0.632	0.931
PAG-rACC	0.081	1	0.776	0.931
PAG-sgACC	0.001	1	0.975	0.975
PAG-dlPFC	0.466	1	0.495	0.931

Supplementary Table 4. Standardized associations between DPMS connectivity and FMI scores by chronic pain status. Standardized β coefficients, 95% confidence intervals (CI), standard errors (SE), Z-values, and P-values are reported from an unconstrained multi-group SEM examining resting-state functional connectivity within the descending pain modulatory system (DPMS). Estimates are stratified by presence of chronic pain. False discovery rate (FDR) correction was applied across all 12 connectivity paths (6 per group) using the Benjamini–Hochberg procedure (Pcorr).

	**β**	**95%CI lower**	**95%CI upper**	**SE**	**Z**	**P**	**P_{corr}**
**Chronic Pain**							
PAG-amygdala	0.024	0.009	0.039	0.008	3.079	0.002	0.012
RVM-PAG	0.000	-0.014	0.014	0.007	-0.010	0.992	0.992
RVM-hypothalamus	-0.012	-0.027	0.003	0.008	-1.626	0.104	0.258
RVM-amygdala	0.016	0.001	0.031	0.008	2.037	0.042	0.167
PAG-hypothalamus	-0.029	-0.043	-0.014	0.007	-3.880	0.0001	0.001
Hypothalamus-Amygdala	-0.007	-0.021	0.008	0.007	-0.912	0.362	0.551
**No Chronic Pain**							
PAG-amygdala	0.002	-0.015	0.019	0.009	0.236	0.814	0.887
RVM-PAG	-0.002	-0.018	0.013	0.008	-0.312	0.755	0.887
RVM-hypothalamus	-0.013	-0.029	0.003	0.008	-1.610	0.107	0.258
RVM-amygdala	0.008	-0.008	0.025	0.008	0.974	0.330	0.551
PAG-hypothalamus	0.007	-0.009	0.023	0.008	0.902	0.367	0.551
Hypothalamus-Amygdala	-0.006	-0.022	0.010	0.008	-0.765	0.445	0.593

Supplementary Table 5. Wald test results comparing connectivity-FMI associations across groups. Wald χ^2 statistics, degrees of freedom, and P-values for each functional connectivity edge within the DPMS. False discovery rate (FDR) correction was applied across all 6 connectivity paths using the Benjamini–Hochberg procedure (Pcorr).

**Edge**	**ChiSq**	**Df**	**P**	**P_{corr}**
PAG-amygdala	6.745	1	0.009	0.028
RVM-PAG	0.020	1	0.889	0.889
RVM-Hypothalamus	0.463	1	0.496	0.744
RVM-Amygdala	1.775	1	0.183	0.365
PAG-Hypothalamus	14.777	1	0.0001	0.001
Hypothalamus-Amygdala	0.199	1	0.655	0.787

Supplementary Table 6. Variance inflation factors (VIFs) for predictors in the fully adjusted mediation model.
VIF values assess multicollinearity between predictors, with values below 5 generally indicating acceptable levels of collinearity. In this model, all predictors showed VIFs well within acceptable limits. The variables included self-reported sleep duration (coded as normal or short/long sleep), pain severity measured using an 11-point numerical rating scale (NRS), depressive symptoms assessed via the Patient Health Questionnaire-9 (PHQ-9), and anxiety symptoms via the Generalized Anxiety Disorder-7 (GAD-7). Cognitive symptoms were captured using an indicator of self-reported brain fog from the symptom severity scale, while fatigue was measured using the Fatigue Severity Scale (FSS). The Widespread Pain Index (WPI) was included as a marker of spatial pain distribution. Additional covariates were age, sex (coded 1 = female), intracranial head size, and scanner table position in the X, Y, and Z dimensions (TablePos_COG_X, Y, Z). Technical and demographic covariates included the scan day (to account for temporal scanner drift), mean relative head motion during resting-state fMRI (HeadMotion_mean_rfMRI_rel), educational attainment (binary indicator for university degree), the Townsend Deprivation Index as a measure of socioeconomic disadvantage, self-reported white ethnicity (binary), body mass index (BMI), and smoking status (binary indicator of current smoking).

**Variable**	**VIF**
Sleep	1.02690945
Pain Severity NRS	1.16067408
PHQ-9	2.09773834
GAD-7	1.30220239
Brain fog	1.33562528
FSS	1.74368583
WPI	1.1559769
Age	1.2239429
Female sex	1.7122419
Head size	1.71121507
TablePos_COG_X	1.0316927
TablePos_COG_Y	1.22218254
TablePos_COG_Z	1.14583375
Scan Day	1.3324525
HeadMotion_mean_rfMRI_rel	1.66444458
University Degree	1.06072337
Townsend Deprivation Index	1.03389863
White ethnicity	1.03540796
BMI	1.65510335
Smoker	1.02423107

Supplementary Table 7. Associations between DPMS functional connectivity and DN4 scores.
Standardized β coefficients, 95% confidence intervals (CI), standard errors (SE), Z-values, and P-values from a structural equation model (SEM) examining the relationship between resting-state functional connectivity within the descending pain modulatory system (DPMS) and neuropathic pain symptoms (DN4 score). False discovery rate (FDR) correction was applied across all 6 connectivity paths using the Benjamini–Hochberg procedure (Pcorr). No connectivity edges were significantly associated with DN4.

Edge	β	95%CI lower	95%CI upper	SE	Z	P	P_{corr}
RVM-PAG	0.009	-0.004	0.023	0.007	1.339	0.181	0.635
PAG-Amygdala	0.004	-0.009	0.018	0.007	0.632	0.527	0.635
PAG-Hypothalamus	0.004	-0.010	0.017	0.007	0.524	0.600	0.635
PAG-rACC	-0.003	-0.017	0.010	0.007	-0.475	0.635	0.635
PAG-sgACC	0.005	-0.009	0.019	0.007	0.715	0.475	0.635
PAG-dlPFC	0.008	-0.005	0.022	0.007	1.226	0.220	0.635

Supplementary Table 8. Associations between DPMS structural connectivity and DN4 scores.
SEM results for structural (diffusion-based) connectivity within the DPMS in relation to DN4 scores. False discovery rate (FDR) correction was applied across all 6 connectivity paths using the Benjamini–Hochberg procedure (Pcorr).

Edge	β	95%CI lower	95%CI upper	SE	Z	P	P_{corr}
RVM-PAG	0.010	-0.005	0.025	0.008	1.291	0.197	0.394
RVM-Hypothalamus	-0.011	-0.027	0.004	0.008	-1.423	0.155	0.394
RVM-Amygdala	0.017	0.001	0.033	0.008	2.093	0.036	0.218
PAG-Hypothalamus	-0.007	-0.022	0.009	0.008	-0.834	0.404	0.606
PAG-Amygdala	0.003	-0.013	0.019	0.008	0.334	0.738	0.823
Hypothalamus-Amygdala	-0.002	-0.017	0.014	0.008	-0.223	0.823	0.823

Supplementary Table 9. Associations between DPMS functional connectivity and pain intensity (NRS).
SEM results for resting-state functional connectivity and self-reported pain intensity (Numerical Rating Scale, NRS). False discovery rate (FDR) correction was applied across all 6 connectivity paths using the Benjamini–Hochberg procedure (Pcorr).

Edge	β	95%CI lower	95%CI upper	SE	Z	P	P_{corr}
RVM-PAG	-0.001	-0.014	0.013	0.007	-0.081	0.935	0.935
PAG-Amygdala	0.011	-0.002	0.025	0.007	1.620	0.105	0.217
PAG-Hypothalamus	0.008	-0.006	0.021	0.007	1.107	0.268	0.402
PAG-rACC	-0.017	-0.031	-0.004	0.007	-2.479	0.013	0.079
PAG-sgACC	0.003	-0.011	0.016	0.007	0.373	0.709	0.851
PAG-dlPFC	0.011	-0.002	0.025	0.007	1.605	0.108	0.217

Supplementary Table 10. Associations between DPMS structural connectivity and pain intensity (NRS).
Standardized SEM results assessing structural connectivity within the DPMS and its relationship with pain intensity (NRS). False discovery rate (FDR) correction was applied across all 6 connectivity paths using the Benjamini–Hochberg procedure (Pcorr).

Edge	β	95%CI lower	95%CI upper	SE	Z	P	P_{corr}

RVM-PAG	-0.005	-0.020	0.010	0.008	-0.640	0.522	0.776
RVM-Hypothalamus	-0.004	-0.020	0.011	0.008	-0.555	0.579	0.776
RVM-Amygdala	0.003	-0.013	0.019	0.008	0.348	0.727	0.776
PAG-Hypothalamus	-0.004	-0.019	0.012	0.008	-0.441	0.659	0.776
PAG-Amygdala	0.002	-0.014	0.019	0.008	0.285	0.776	0.776
Hypothalamus-Amygdala	0.009	-0.006	0.025	0.008	1.150	0.250	0.776

Supplementary Table 11. Standardized associations between DPMS functional connectivity and FMI scores by age-group in adults with chronic pain.
Standardized β coefficients, 95% confidence intervals (CI), standard errors (SE), Z-values, and P-values are reported from an unconstrained multi-group SEM examining resting-state functional connectivity within the descending pain modulatory system (DPMS). Estimates are stratified by 5-year age band. An overall group difference in connectivity effects was observed (likelihood ratio test P = 0.023).

	**group (years)**	**N**	**β**	**95%CI lower**	**95%CI upper**	**SE**	**Z**	**P**	**P_{corr}**
RVM-PAG	40-45	3,054	0.020	-0.010	0.049	0.015	1.282	0.200	0.688
PAG-Amygdala	40-45		0.025	-0.006	0.055	0.015	1.600	0.110	0.507
PAG-Hypothalamus	40-45		-0.006	-0.036	0.024	0.015	-0.365	0.715	0.860
PAG-rACC	40-45		0.003	-0.027	0.034	0.015	0.224	0.823	0.872
PAG-sgACC	40-45		0.017	-0.013	0.047	0.015	1.116	0.264	0.699
PAG-dlPFC	40-45		0.043	0.013	0.073	0.015	2.814	0.005	0.059
RVM-PAG	46-50	3,829	-0.009	-0.036	0.017	0.013	-0.680	0.497	0.797
PAG-Amygdala	46-50		0.017	-0.010	0.043	0.014	1.253	0.210	0.688
PAG-Hypothalamus	46-50		0.021	-0.005	0.048	0.013	1.586	0.113	0.507
PAG-rACC	46-50		-0.025	-0.051	0.002	0.014	-1.809	0.070	0.487
PAG-sgACC	46-50		0.005	-0.022	0.031	0.014	0.354	0.724	0.860
PAG-dlPFC	46-50		0.011	-0.016	0.037	0.014	0.803	0.422	0.797
RVM-PAG	51-55	4,586	-0.012	-0.046	0.021	0.017	-0.711	0.477	0.797
PAG-Amygdala	51-55		0.024	-0.010	0.058	0.017	1.396	0.163	0.651
PAG-Hypothalamus	51-55		-0.016	-0.050	0.018	0.017	-0.935	0.350	0.699
PAG-rACC	51-55		-0.016	-0.050	0.018	0.017	-0.937	0.349	0.699
PAG-sgACC	51-55		-0.016	-0.050	0.018	0.017	-0.942	0.346	0.699
PAG-dlPFC	51-55		-0.007	-0.041	0.027	0.017	-0.414	0.679	0.860
RVM-PAG	56-60	5,148	0.025	-0.023	0.074	0.025	1.019	0.308	0.699
PAG-Amygdala	56-60		-0.008	-0.058	0.041	0.025	-0.334	0.738	0.860
PAG-Hypothalamus	56-60		0.043	-0.005	0.092	0.025	1.744	0.081	0.487
PAG-rACC	56-60		-0.008	-0.058	0.041	0.025	-0.331	0.740	0.860
PAG-sgACC	56-60		0.024	-0.025	0.074	0.025	0.961	0.337	0.699
PAG-dlPFC	56-60		0.016	-0.034	0.065	0.025	0.626	0.532	0.797
RVM-PAG	61-65	4,060	0.008	-0.023	0.038	0.016	0.484	0.628	0.860
PAG-Amygdala	61-65		-0.007	-0.037	0.024	0.016	-0.422	0.673	0.860
PAG-Hypothalamus	61-65		0.011	-0.020	0.041	0.016	0.686	0.493	0.797
PAG-rACC	61-65		0.016	-0.015	0.047	0.016	1.019	0.308	0.699
PAG-sgACC	61-65		0.003	-0.027	0.034	0.016	0.223	0.823	0.872
PAG-dlPFC	61-65		0.001	-0.030	0.031	0.016	0.037	0.970	0.970
RVM-PAG	66-70	1,537	0.009	-0.019	0.037	0.014	0.643	0.520	0.797
**PAG-Amygdala**	**66-70**		**0.046**	**0.018**	**0.075**	**0.014**	**3.237**	**0.001**	**0.022**
PAG-Hypothalamus	66-70		0.004	-0.024	0.032	0.014	0.252	0.801	0.872
PAG-rACC	66-70		-0.002	-0.031	0.026	0.014	-0.163	0.870	0.895
PAG-sgACC	66-70		0.026	-0.002	0.054	0.014	1.814	0.070	0.487
**PAG-dlPFC**	**66-70**		**-0.053**	**-0.081**	**-0.025**	**0.014**	**-3.687**	**0.000**	**0.008**

Supplementary Table 12. Wald test results comparing functional connectivity-FMI associations across age groups.
Wald χ^2 statistics, degrees of freedom, and P-values for each functional connectivity edge within the DPMS. False discovery rate (FDR) correction was applied across all 6 connectivity paths using the Benjamini–Hochberg procedure (Pcorr). An overall group difference in connectivity effects was observed (likelihood ratio test P = 0.023).

**Edge**	**ChiSq**	**Df**	**P**	**P_{corr}**
RVM-PAG	3.648	5	0.601	0.601
PAG-Amygdala	7.841	5	0.165	0.496
PAG-Hypothalamus	5.702	5	0.336	0.601
PAG-rACC	4.612	5	0.465	0.601
PAG-sgACC	4.044	5	0.543	0.601
**PAG-dlPFC**	**22.628**	**5**	**0.0004**	**0.002**

Supplementary Table 13. Standardized associations between DPMS structural connectivity and FMI scores by age-group in adults with chronic pain.
Standardized β coefficients, 95% confidence intervals (CI), standard errors (SE), Z-values, and P-values are reported from an unconstrained multi-group SEM examining structural connectivity within the descending pain modulatory system (DPMS). Estimates are stratified by 5-year age band. No overall group difference in connectivity effects was observed (likelihood ratio test P = 0.342).

	**group (years)**	**β**	**95%CI lower**	**95%CI upper**	**SE**	**Z**	**P**	**P_{corr}**
RVM-PAG	40-45	0.024	0.018	1.364	-0.010	0.058	0.172	0.510
RVM-Hypothalamus	40-45	-0.033	0.016	-2.044	-0.065	-0.001	0.041	0.295
RVM-Amygdala	40-45	-0.017	0.017	-0.974	-0.051	0.017	0.330	0.699
PAG-Hypothalamus	40-45	0.011	0.018	0.623	-0.024	0.046	0.533	0.711
PAG-Amygdala	40-45	-0.012	0.018	-0.710	-0.047	0.022	0.478	0.711
Hypothalamus-Amygdala	**40-45**	-0.012	0.017	-0.684	-0.044	0.021	0.494	0.711
RVM-PAG	46-50	0.017	0.016	1.086	-0.014	0.049	0.277	0.624
RVM-Hypothalamus	46-50	0.000	0.015	-0.028	-0.029	0.028	0.978	0.990
RVM-Amygdala	46-50	-0.013	0.016	-0.855	-0.044	0.017	0.393	0.711
PAG-Hypothalamus	46-50	0.028	0.016	1.738	-0.004	0.060	0.082	0.346
PAG-Amygdala	46-50	-0.024	0.015	-1.542	-0.054	0.006	0.123	0.443
Hypothalamus-Amygdala	46-50	0.003	0.015	0.179	-0.027	0.032	0.858	0.929
RVM-PAG	51-55	0.030	0.022	1.368	-0.013	0.072	0.171	0.510
RVM-Hypothalamus	51-55	0.012	0.019	0.663	-0.024	0.049	0.507	0.711
RVM-Amygdala	51-55	-0.004	0.020	-0.211	-0.044	0.036	0.833	0.929
PAG-Hypothalamus	51-55	0.015	0.021	0.732	-0.025	0.056	0.464	0.711
PAG-Amygdala	51-55	-0.037	0.020	-1.886	-0.076	0.001	0.059	0.346
Hypothalamus-Amygdala	51-55	-0.044	0.020	-2.189	-0.084	-0.005	0.029	0.257
RVM-PAG	56-60	0.062	0.028	2.234	0.008	0.117	0.025	0.257
RVM-Hypothalamus	56-60	-0.007	0.027	-0.246	-0.059	0.046	0.806	0.929
RVM-Amygdala	56-60	0.049	0.028	1.718	-0.007	0.105	0.086	0.346
PAG-Hypothalamus	56-60	0.024	0.031	0.780	-0.036	0.084	0.436	0.711
PAG-Amygdala	56-60	-0.037	0.028	-1.328	-0.092	0.018	0.184	0.510
Hypothalamus-Amygdala	56-60	-0.029	0.027	-1.087	-0.082	0.023	0.277	0.624
RVM-PAG	61-65	0.047	0.019	2.409	0.009	0.085	0.016	0.257
RVM-Hypothalamus	61-65	0.020	0.017	1.167	-0.013	0.053	0.243	0.624
RVM-Amygdala	61-65	-0.015	0.018	-0.821	-0.051	0.021	0.412	0.711
PAG-Hypothalamus	61-65	0.003	0.019	0.155	-0.034	0.040	0.877	0.929
PAG-Amygdala	61-65	-0.039	0.018	-2.220	-0.074	-0.005	0.026	0.257
Hypothalamus-Amygdala	61-65	0.006	0.018	0.305	-0.030	0.041	0.761	0.913
RVM-PAG	66-70	-0.007	0.017	-0.379	-0.040	0.027	0.705	0.875
RVM-Hypothalamus	**66-70**	0.000	0.015	0.012	-0.030	0.030	0.990	0.990
RVM-Amygdala	66-70	-0.028	0.016	-1.714	-0.061	0.004	0.086	0.346
PAG-Hypothalamus	66-70	0.011	0.017	0.643	-0.022	0.044	0.520	0.711
PAG-Amygdala	66-70	-0.015	0.016	-0.901	-0.046	0.017	0.368	0.711
Hypothalamus-Amygdala	66-70	0.009	0.016	0.551	-0.023	0.041	0.582	0.748

Supplementary Table 14. Wald test results comparing structural connectivity-FMI associations across age groups.
Wald χ^2 statistics, degrees of freedom, and P-values for each structural connectivity edge within the DPMS. False discovery rate (FDR) correction was applied across all 6 connectivity paths using the Benjamini–Hochberg procedure (Pcorr). No overall group difference in connectivity effects was observed (likelihood ratio test P = 0.451).

**Edge**	**ChiSq**	**Df**	**P**	**P_{corr}**
RVM-PAG	6.553	5	0.256	0.477
RVM-Hypothalamus	5.985	5	0.308	0.477
RVM-Amygdala	5.881	5	0.318	0.477
PAG-Hypothalamus	1.154	5	0.949	0.949
PAG-Amygdala	2.993	5	0.701	0.841
Hypothalamus-Amygdala	6.103	5	0.296	0.477

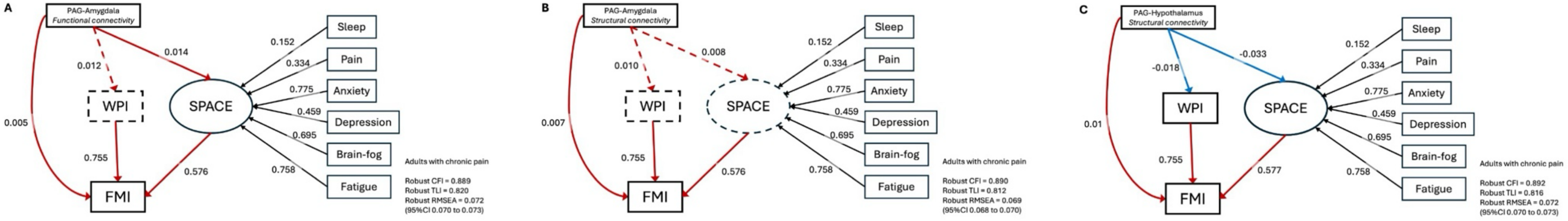

A

Path	Estimate	CI Lower	CI Upper	P (uncorrected)	P (corrected)
Indirect pathways					
SPACE	0.008	0.0002	0.016	0.042	0.067
Widespread Pain	0.009	-0.0003	0.019	0.067	0.067
Total Indirect	**0.017**	0.004	0.032	0.02	
Direct	**0.005**	-0.0007	0.009	0.056	
Total	**0.022**	0.008	0.036	0.002	

B

Path	Estimate	CI Lower	CI Upper	P (uncorrected)	P (corrected)
Indirect pathways					
SPACE	0.004	-0.004	0.017	0.296	0.296
Widespread Pain	0.007	-0.004	0.023	0.166	0.296
Total Indirect	**0.012**	-0.004	0.036	0.141	
Direct	**0.007**	0.002	0.015	0.006	
Total	**0.019**	0.005	0.043	0.018	

C

Path	Estimate	CI Lower	CI Upper	P (uncorrected)	P (corrected)
Indirect pathways					
SPACE	-0.019	-0.032	-0.012	0.000014	0.000028
Widespread Pain	-0.014	-0.028	-0.005	0.01	0.01
Total Indirect	**-0.033**	-0.057	-0.020	0.00006	
Direct	**0.010**	0.006	0.018	0.0002	
Total	**-0.023**	-0.044	-0.009	0.0037	

Supplementary Figure 2. Mediation models using a latent SPACE symptom factor to explain associations between DPMS connectivity and nociplastic pain severity. Structural equation models were used to evaluate whether a latent SPACE symptom factor – reflecting shared variance among Sleep disturbance, Pain, Affect (depression/anxiety), Cognitive symptoms, and low Energy – along with Widespread Pain Index (WPI), mediated the associations between Descending Pain Modulatory System (DPMS) connectivity and Fibromyalgia Index (FMI) scores in adults with chronic pain. Each panel depicts a separate mediation model: (A) PAG-amygdala functional connectivity; (B) PAG-amygdala structural connectivity; and (C) PAG-hypothalamus structural connectivity. Arrows indicate directional associations, from brain connectivity metrics to mediators, and from mediators to FMI, along with the direct brain–FMI path. Standardised path coefficients are shown. Red arrows indicate positive associations; blue arrows indicate negative associations. Solid lines denote significant paths ($P < 0.05$); dashed lines indicate non-significant paths. Tables below each model summarize total, direct, and indirect effects. Model fit indices (Robust CFI, TLI, RMSEA) suggest good to excellent fit across models. PAG = periaqueductal grey; FMI = Fibromyalgia Index; WPI = Widespread Pain Index.
