# Supplementary material for: Brain signatures of nociplastic pain: Fibromyalgia Index and descending modulation at population level
Source: Brain. Author manuscript; Available in PMC 2026 Feb 5. (PMC7618718; doi:10.1093/brain/awaf307)
Supplement: Supplementary file 1 [file EMS212269-supplement-Author_contributions.pdf]

Author contributions

EK, AS and AI conceived and designed the analysis, collected the data, contributed data or analysis tools, performed the analysis, and wrote the paper. EK, AS and AI directly accessed and verified the data. VW, AS, FL, TRM, TN, BS, and IT contributed by conceiving and designing the analysis and writing the paper. All authors approved the final version of the manuscript.
